# Efficacy and safety of conduction system pacing in heart failure patients with non-left bundle branch block morphology: a systematic review and meta-analysis

**DOI:** 10.3389/fphys.2025.1716337

**Published:** 2025-11-17

**Authors:** Kaung Myat Thu, Ibrahim Antoun, Mahmoud Eldesouky, Ahmed Abdelrazik, May Myat Thaint, G. André Ng, Mokhtar Ibrahim

**Affiliations:** 1 Department of Cardiology, University Hospitals of Leicester NHS Trust, Glenfield Hospital, Leicester, United Kingdom; 2 Department of Cardiovascular Sciences, Clinical Science Wing, University of Leicester, Glenfield Hospital, Leicester, United Kingdom; 3 Respiratory Department, Darlington Memorial Hospital, Darlington, United Kingdom

**Keywords:** conduction system pacing, His bundle pacing, left bundle branch area pacing, cardiac resynchronisation therapy, biventricular pacing, heart failure, non-left bundle branch block

## Abstract

**Background:**

Conduction system pacing, including His bundle pacing and left bundle branch area pacing, has emerged as a physiological alternative to biventricular pacing (BiVP) for cardiac resynchronisation therapy (CRT). BiVP benefits patients with left bundle branch block (LBBB), but outcomes in non-LBBB morphologies are inconsistent. We synthesised the evidence for CSP in heart failure patients with non-LBBB conduction patterns.

**Methods:**

We performed a systematic review and meta-analysis (PROSPERO CRD420251015905) of 21 studies (11 with quantitative data; n = 480). Comparative outcomes (CSP vs. BiVP) and baseline vs. follow-up CSP changes were pooled. Primary endpoints were left ventricular ejection fraction (LVEF), left ventricular end-diastolic diameter (LVEDD), New York Heart Association (NYHA) class, and QRS duration. Secondary endpoints included heart failure hospitalisation and all-cause mortality.

**Results:**

In head-to-head analyses (198 patients; 99 per arm), CSP conferred a mean + 5.83% LVEF benefit (95% CI 3.06–8.60; *p* < 0.0001; I^2^ = 0%), reduced LVEDD by 3.87 mm (95% CI 2.53–5.21; *p* < 0.001), improved NYHA class by −0.30 (95% CI –0.46 to −0.13; *p* = 0.0004), and narrowed QRS (SMD –0.91; 95% CI –1.18 to −0.64; *p* < 0.00001). CSP also halved HF hospitalisation risk (RR 0.44; 95% CI 0.24–0.81; *p* = 0.008; I^2^ = 0%). In single-arm baseline and follow-up analyses (480 patients), CSP yielded a mean + 8.91% LVEF, −2.95 mm LVEDD, SMD –1.37 NYHA, and SMD –1.21 QRS (*p* < 0.0001).

**Conclusion:**

In non-LBBB heart failure, CSP delivers substantial improvements in ventricular systolic function, reverse remodelling, symptoms, and electrical synchrony versus BiVP, with reduced HF hospitalisation. These findings position CSP as a promising BiVP strategy for a traditionally non-responder subgroup and warrant confirmation in large, randomised trials.

## Graphical Abstract

**Figure:**
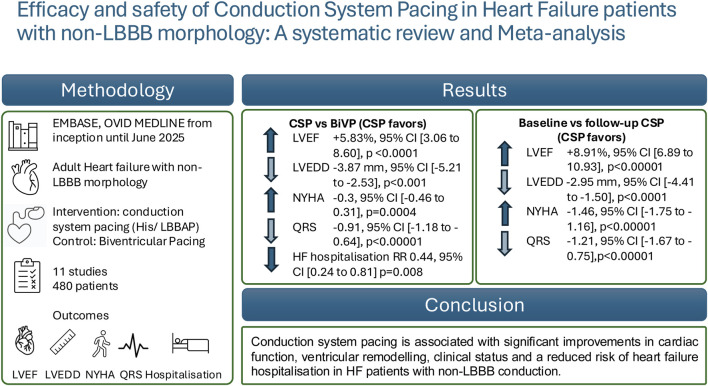
Infographic Abstract of the meta-analysis of efficacy and safety of conduction system pacing in Heart Failure patients with non-left bundle branch block morphology.

## Introduction

Cardiac resynchronisation therapy (CRT) with biventricular pacing (BiVP) is an established treatment for patients with heart failure and left bundle branch block (LBBB), improving symptoms, ventricular function, and survival (Glikson et al., 2021). However, the clinical benefit of BiVP in patients with non-LBBB conduction disturbances is less consistent, with multiple trials reporting attenuated or absent improvements in this subgroup. Consequently, guideline recommendations for CRT in non-LBBB patients remain cautious, and optimal pacing strategies for this population are not well defined (Glikson et al., 2021).

Conduction system abnormalities in patients with heart failure with reduced ejection fraction frequently exceed the classical left bundle branch block, including right bundle branch block (RBBB), intraventricular conduction delay (IVCD), and nonspecific QRS prolongation. These conduction patterns, collectively referred to as non-LBBB morphologies, typically present with a QRS duration exceeding 120 milliseconds yet lack the characteristic left-sided delay seen in LBBB. Right bundle branch block is electrocardiographically characterised by an rsr', rsR', or rSR' pattern in leads V1 or V2, with a QRS duration of no less than 120 m. The terminal R wave is commonly broader than the initial R, with a prolonged S wave in leads I and V6. Atypical RBBB can exhibit a wide, notched R wave in leads I and aVL, regularly with a left axis deviation. In contrast, intraventricular conduction delay is a nonspecific conduction slowing where QRS widening does not meet the standards for either typical LBBB or RBBB and may display discordant conduction patterns between limb and precordial leads.

Conduction system pacing (CSP), achieved through His bundle pacing (HBP) or left bundle branch area pacing (LBBAP), has emerged as an alternative resynchronisation strategy designed to restore physiological ventricular activation by directly engaging the His–Purkinje network. Early studies suggest that CSP may provide superior electrical and mechanical synchrony compared with BiVP, potentially translating into improved functional and structural outcomes, particularly in patients with non-LBBB conduction patterns who respond poorly to conventional CRT (Sharma et al., 2018).

Although several studies have evaluated CSP in this setting, individual trials are limited by small sample sizes, heterogeneous methodologies, and variable endpoints. A systematic synthesis of available evidence is therefore required to clarify the effectiveness of CSP relative to BiVP in non-LBBB heart failure and to evaluate its impact on key clinical, echocardiographic, and electrophysiological outcomes.

Accordingly, we conducted a systematic review and meta-analysis to assess the comparative and pooled effects of CSP versus BiVP on left ventricular function, reverse remodelling, symptom status, and QRS duration in patients with non-LBBB conduction patterns.

## Methodology

The steps involved in conducting this systematic review are reported in accordance with the Preferred Reporting Items for Systematic Reviews and Meta-Analyses (PRISMA) statement standard (Moher et al., 2009). A study protocol following the PRISMA guidelines was previously registered at the International Prospective Register of Systematic Reviews (PROSPERO ID CRD420251015905) with the PRISMA checklist available in our Supplementary Material.

Statistical analysis was carried out by the RevMan 5.4 using the random effect model (RevMan, 2020). Continuous variables were combined utilising the standard mean difference with a 95% confidence interval CI. I^2^ was calculated to assess statistical heterogeneity, and I^2^ >50% was described as substantial heterogeneity. Funnel plots were drawn with standard deviation to visually determine publication bias. For single-arm pre–post analyses, we synthesised changes in outcomes from baseline to follow-up. Because correlation coefficients were rarely reported, pooled standardised mean differences (SMDs) were calculated under the assumption of independence. This approach may underestimate variance and overstate precision. To mitigate this, we conducted sensitivity analyses using reported change scores when available, and we emphasise that these findings are exploratory rather than confirmatory.

### Search strategy

An electronic search was conducted of EMBASE and OVID MEDLINE for studies containing Medical Subject Headings (MeSH) terms or keywords related to *conduction system pacing*, *His bundle pacing*, *left bundle branch area pacing*, *biventricular pacing*, *cardiac resynchronization therapy*, *heart failure*, and *non–left bundle branch block (non-LBBB)* conduction patterns from inception until June 2025 (Supplementary Material provides complete search strategy). No language restrictions were applied during the initial search. Studies were eligible if they reported on adult patients with heart failure and non-LBBB conduction disturbances and included outcomes relating to left ventricular function, structural remodelling, symptom status, or QRS duration.

We excluded case reports, conference abstracts without full-text availability, studies without quantitative outcome data, and those involving only patients with typical LBBB. Relevant articles were also identified through manual screening of the reference lists of prior systematic reviews and meta-analyses.

We only included papers published in English with full text for further analysis. K.M.T, I.A (reviewer) searched databases. Data was imported to the Rayyan QCRI (web app) (Ouzzani et al., 2016) for title/abstract screening and subsequent removal of duplicates.

From exclusion to inclusion, the titles and abstracts of papers identified through the electronic search were examined to select relevant documents. Four reviewers independently evaluated it. Disagreements were resolved through consensus discussions among the three reviewers. Any studies not excluded at this stage were then reviewed in full to see if they met the inclusion criteria. The final conflicts were resolved by consensus discussion with three reviewers who had developed and tested the study selection criteria.

### Study eligibility criteria

We included randomised control trials and observational comparative studies comparing the effectiveness of CSP with BiVP in delivering CRT, after excluding duplicates. Case Reports, Literature Reviews, and Editorial/Meta-analysis were excluded. In our study, the characteristics of the study population were adults (>18 years of age) with heart failure, right bundle branch block, and intraventricular conduction delay.

### Data extraction and quality assessment

We devised a standardised form to extract data from the eligible studies to evaluate study quality and evidence synthesis. The data analysis was conducted using the Excel 2016 version (Microsoft, Redmond, WA, United States). Data extraction was performed from figures, tables, graphs, and the main text. Five reviewers collected data parameters (if reported) using the purpose-designed data collection sheet. These data (where reported) were categorised under the following headings for all included studies: Title, Author, Year, Journal, Study Design, Population Demographics and Baseline QRS/Left ventricular ejection fraction (LVEF)/Left ventricular end diastolic diameter (LVEDD)/New York Heart Association (NYHA) class; Paced QRS duration; Follow-up NYHA class/Echo/LVEDD. Methodological quality of included literature was assessed by the above researchers using the Joanna Briggs Institute (JBI) tool (Munn et al., 2020) for observational studies and additional assessment as risk of bias assessment version 2 (ROB-S) (RoB 2, 2020) for randomised trials.

## Results

### Study selection

We conducted a systematic review and meta-analysis of studies comparing conduction system pacing, including His bundle pacing, left bundle branch area pacing, with biventricular pacing in patients with heart failure and non-LBBB conduction patterns. Eligible studies were required to report at least one of the following outcomes: LVEF, LVEDD, NYHA functional class, or QRS duration. Both randomised controlled trials and observational cohort studies were considered. Studies were excluded if they lacked a comparator arm, enrolled patients exclusively with LBBB, or did not provide extractable quantitative data.

The initial search and screening process yielded a pool of studies, of which 11 met the inclusion criteria for LVEF analysis, seven for LVEDD, eight for NYHA functional class, and eight for QRS duration. For direct head-to-head comparisons between CSP and BiVP, three studies contributed data on LVEF, three on LVEDD, two on NYHA class, and three on QRS duration.

### Study characteristics

A total of 11 unique studies involving 480 patients were included across the different outcome analyses. Study designs included both prospective randomised trials and retrospective observational cohorts, with sample sizes ranging from fewer than 20 to over 100 patients. The mean follow-up period varied from 6 to 24 months Table 1 *.*

**TABLE 1:** Baseline characteristics of included studies.

Study	Year	Type of study	Intervention	ECG morphology	Participants	Mean ages (years)	Sex (male)	Diabetes	Hypertension	LVEF (baseline)	LVEDD (baseline)	NYHA class	QRS duration
Vijayaraman et al. (2021)	2021	Observational, Feasibility	LBBAP	Non-LBBB	116/227	71 ± 12	176/227	100/227	188/227	33 ± 10	57 ± 10	2.7 ± 0.7	160 ± 28
Bednarek et al. (2023)	2023	Observational	LBBAP	RBBB, IVCD	39/101	76.1 ± 11.8	50/101	35/101	84/101	41.4 ± 9.2			
Tan et al. (2023)	2023	Observational	CSP BiVP	Non-LBBB	48 48	70 ± 11	75/96	53/96	70/96	29 ± 9	56 ± 8		158 ± 26
Huang et al. (2022)	2022	Randomised control trial	HBP BiVP	RBBB, narrow QRS	25 25	64.3	36	7	32	32.6 ± 6.4	62.5 ± 8.5	2.8 ± 0.6	
Chen et al. (2023)	2023	Observational	LOT-CRT BiV-CRT	IVCD	30 55	63.7 ± 13.3	61/85	25/85	24/85	30 ± 7.3	62.5 ± 9.5	2.6 ± 0.5	183.6 ± 20
Vijayaraman et al. (2022)	2022	Observational Feasibility	LBBAP	RBBB	107	74 ± 12	81	40	80	35 ± 9	54 ± 12	2.5 ± 0.8	156 ± 20
Ma et al. (2023)	2023	Observational, Feasibility	CSP	Non-LBBB	18/70	66 ± 8.84	39/70	18/70	32/70	24.9 ± 1.95	63.7 ± 7.8	3.2 ± 0.5	139 ± 43
Tam et al. (2024)	2024	Observational, Feasibility	CSP	RBBB, IVCD	20	69 ± 11	18	10	13	26 ± 6		3 ± 0.5	165 ± 19
Gardas et al. (2024)	2022	Observational	HBP	IVCD	14	66.1 ± 9.55	12	5	8	28.8 ± 6.84		2.6 ± 0.6	162.1 ± 14.7
Sharma et al. (2018)	2018	Observational, Feasibility	HBP	RBBB	37	72 ± 10	33	10	35	31 ± 10	57 ± 7	2.8 ± 0.6	158 ± 24
Upadhyay et al. (2021)	2021	Observational	HBP	RBBB, narrow QRS	30	68 ± 15	22	10	23	31 ± 6			

Values are mean ±, SD, or n (%).

BiV-CRT, BiVP, biventricular pacing; CSP, conduction system pacing; HBP, his bundle pacing; IVCD, intraventricular conduction delay; LOT-CRT, left bundle branch-optimised cardiac resynchronisation therapy; LBBAP, left bundle branch area pacing; LVEDD, left ventricular end diastolic diameter; LVEF, left ventricular ejection fraction; non-LBBB, non-left bundle branch block; NYHA, class = New York Heart Association class; RBBB, right bundle branch block.

Across studies, CSP was delivered using either HBP or left bundle branch area pacing (LBBAP), with technique selection based on operator preference and anatomical feasibility. BiVP was performed using the standard coronary sinus. Patient populations predominantly included individuals with reduced LVEF and non-LBBB conduction disturbances (e.g., right bundle branch block, intraventricular conduction delay). Baseline QRS durations and LVEF values varied across studies, reflecting real-world heterogeneity.

Outcomes were assessed using standard echocardiographic measures (LVEF, LVEDD), NYHA functional classification, and surface electrocardiography for QRS duration. Table 2. Several studies reported procedural success rates and complication profiles, though these were not uniformly available for quantitative synthesis.

**TABLE 2:** Outcomes of included studies.

Study	Year	Intervention	Follow-up (months)	Procedure time (min)	Fluoroscopy time (min)	LVEF (%)	LVEDD (mm)	NYHA class	Paced QRS duration	All-cause mortality	HF hospitalisation	Death or HF hospitalisation	Complications
Vijayaraman et al.	2021	LBBAP	12	105 ± 54	19 ± 15	43 ± 12	55 ± 9	1.8 ± 0.7	143 ± 23	11/227	15/227		Pneumothorax 3, Device infection 2, Leads dislodgement 7
Bednarek et al.	2023	LBBAP	23			45.6 ± 9.9							
Tan et al.	2023	CSP	24		22 ± 17	39 ± 13	52 ± 8		127 ± 28	5/48	9/48		
		BiVP				32 ± 11	59 ± 10		149 ± 16	19/48	18/48		
Huang et al.	2022	HBP	18			37 ± 9.5	55 ± 9.5	1.5 ± 0.6	107.6 ± 12.5	7/50	1/25		Nil
		BiVP				30.5 ± 7	54.6 ± 7	1.58 ± 0.64	135.7 ± 16.6		2/25		
Su et al.	2023	LOT-CRT	24	126.5 ± 22.6		53.9 ± 11.9	56.2 ± 11.6	2.2 ± 0.5	140.9 ± 17.6			2/30 (LOT-CRT)	
		BiV-CRT		105.8 ± 18.1		51.3 ± 7.4	62.5 ± 9.5	2.6 ± 0.6	154.1 ± 20.2			14/55 (BIV-CRT)	
Vijayaraman et al.	2022	LBBAP	13 ± 8	97 ± 48	16 ± 12	43 ± 12	52 ± 12	1.7 ± 0.8	150 ± 24	8/107	9/107		
		BiVP			16.8 ± 7.6	38 ± 6		2.5 ± 0.5	95 ± 15				
Ma et al.	2023	CSP	23.43 ± 11.44			34.6 ± 8.9	55 ± 9.5	1.8 ± 0.8	113 ± 18	13/70	0.53 ± 0.28*		
Tam et al.	2024	CSP	6			34 ± 11			132 ± 20	2/20			Nil
Gardas et al.	2022	HBP	11.7 ± 6.9			33.6 ± 7.55		1.9 ± 0.5	131.4 ± 15.6		4/81		Lead dislodgement1
Vijayaraman et al.	2018	HBP	15 ± 23			39 ± 13	56 ± 10	2 ± 0.7	127 ± 17	2/37	2/37		
Upadhyay et al.	2021	HBP	17 ± 9			40 ± 5.5				2/30	8/30		

Values are mean ± SD or n (%); *Mean.

BiV-CRT, BiVP, biventricular pacing; CSP, conduction system pacing; HBP, his bundle pacing; IVCD, intraventricular conduction delay; LOT-CRT, left bundle branch-optimised cardiac resynchronisation therapy; LBBAP, left bundle branch area pacing; LVEDD, left ventricular end diastolic diameter; LVEF, left ventricular ejection fraction; non-LBBB, non-left bundle branch block; NYHA, class = New York Heart Association class; RBBB, right bundle branch block.

### Statistical analysis

Meta-analyses were performed using standardised mean difference (SMD), or mean difference (MD), with corresponding 95% confidence intervals (CI) for continuous outcomes and risk ratio (RR) with 95% confidence intervals for dichotomous outcomes. When studies reported median and interquartile range, values were converted to mean and standard deviation using validated statistical methods. Pooled estimates were calculated with both fixed-effect and random-effects models, though the latter was prioritised in the presence of heterogeneity.

**FIGURE 1:**
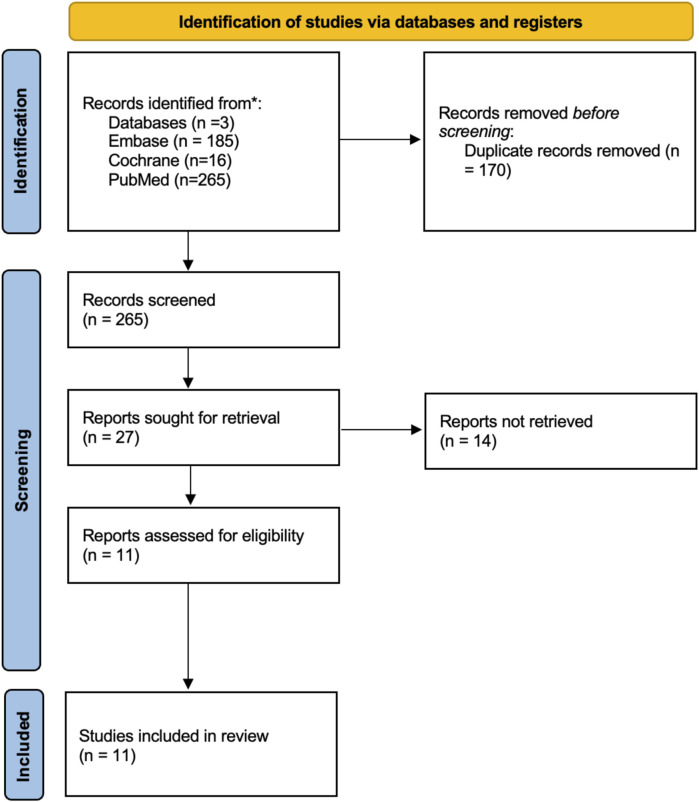
PRISMA flow diagram.

Heterogeneity was assessed using the I^2^ statistic and the Chi^2^ test. Statistical heterogeneity was assessed using the I^2^ statistic. Values of <40% were considered to indicate low heterogeneity, 40%–75% moderate heterogeneity, and >75% considerable heterogeneity. In such cases, potential sources of variability were explored, including differences in pacing modality (HBP vs. LBBAP), baseline ventricular size, and follow-up duration. Forest plots were generated to display individual and pooled effect sizes visually. Sensitivity analyses were performed where possible to evaluate the robustness of findings. Publication bias was not formally assessed due to the limited number of studies per outcome (<10 in most analyses). The PRISMA flow diagram is shown in Figure 1.

### Effect on left ventricular ejection fraction

**FIGURE 2:**
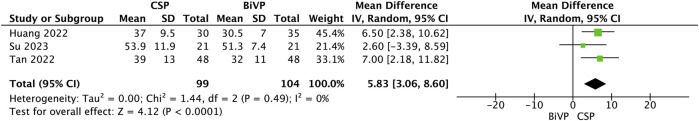
Forest plot showing a comparison of follow up LVEF between conduction system pacing (CSP) and Biventricular pacing (BiVP) for standard mean difference using random effects model.

Three studies (Huang 2022; Sun 2022; Tan 2022) including 198 patients (99 CSP, 99 BiVP) directly compared the effect of conduction system pacing (CSP) and biventricular pacing (BiVP) on LVEF. The pooled analysis demonstrated a significant improvement in LVEF with CSP, with a mean difference of 5.83% (95% CI, 3.06–8.60; *Z* = 4.12; *p* < 0.0001). All three studies consistently favored CSP, with individual mean differences ranging from 6.5% to 7.8%. Statistical heterogeneity was negligible (I^2^ = 0%, χ^2^ = 1.44, *df* = 2, *p* = 0.49), indicating excellent consistency across studies. These findings suggest that CSP provides a clinically meaningful enhancement in systolic function compared with BiVP in non-LBBB patients Figure 2.

A meta-analysis of 11 studies including a total of 480 patients undergoing conduction system pacing assessed changes in left ventricular ejection fraction from baseline to follow-up. The pooled mean difference in LVEF was 8.91% (95% confidence interval [CI], 6.89–10.93), indicating a significant improvement in ventricular function after CSP (*Z* = 6.83, *P* < 0.00001).

Although the overall effect was robust, moderate heterogeneity was observed among the included studies (I^2^ = 63%, τ^2^ = 6.99, χ^2^ = 26.95, *df* = 10, *P* = 0.003), reflecting some variability in magnitude of LVEF improvement across different cohorts and study designs. Individually, studies such as Bednarek et al. (2023), Gardas et al. (2023), and Tam et al. (2024) reported larger mean LVEF increases, while others showed more modest gains. Despite this heterogeneity, the consistent direction of effect supports that CSP reliably improves cardiac function over time.

**FIGURE 3:**
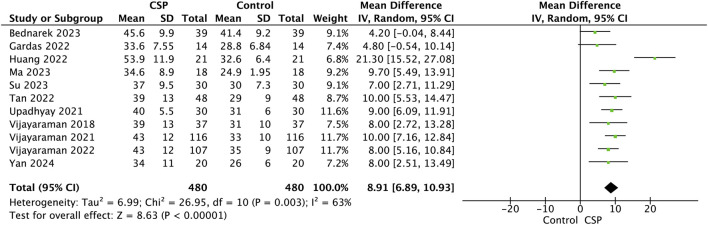
Forest plot showing a comparison of follow-up LVEF between baseline and follow-up conduction system pacing (CSP) implant for the mean difference using a random effects model.

These findings underscore the clinical utility of CSP in enhancing myocardial performance in patients with heart failure, as evidenced by a statistically and clinically meaningful increase in LVEF from baseline to follow-up Figure 3.

### Subgroup analyses (LBBAP, HBP)

**FIGURE 4:**
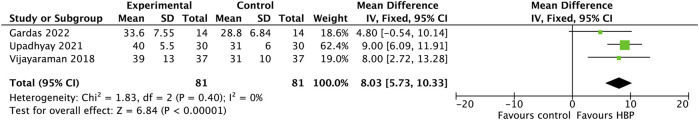
Forest plot showing a comparison of follow-up LVEF between baseline and follow-up His Bundle Pacing implant for the mean difference using a random effects model.

A pooled analysis of three studies comparing LVEF before and after His bundle pacing demonstrated a significant improvement in LVEF following pacing. The mean difference was 8.03% (95% confidence interval [CI] 5.73 to 10.33; p < 0.00001), indicating a consistent positive effect across studies. There was no evidence of statistical heterogeneity (I^2^ = 0%, p = 0.40), supporting the robustness and homogeneity of the observed improvement. Individually, each study showed a numerical increase in LVEF after His bundle pacing, with effect sizes ranging from 4.8% to 9%. Overall, these findings suggest that His bundle pacing is associated with a clinically and statistically significant enhancement in left ventricular systolic function compared with pre-implant values Figure 4.

**FIGURE 5:**
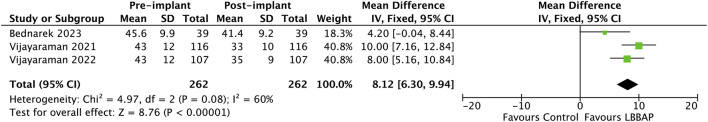
Forest plot showing a comparison of follow-up LVEF between baseline and follow-up Left Bundle Branch Area pacing implant for the mean difference using a random effects model.

The meta-analysis of studies assessing LVEF before and after LBBAP revealed a consistent improvement across all included cohorts. The pooled mean difference was 8.12% (95% CI 6.30 to 9.94; p < 0.00001), indicating a significant enhancement in systolic performance following implantation. Although the degree of heterogeneity was moderate (I^2^ = 60%, p = 0.08), the direction of effect remained uniform, reflecting a reproducible functional gain with LBBAP. These results suggest that LBBAP confers a meaningful recovery of left ventricular contractility, likely driven by more physiological resynchronisation of ventricular activation compared with pre-implant conduction Figure 5.

### Decrease in left ventricular end-diastolic diameter

**FIGURE 6:**
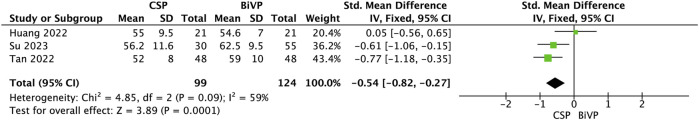
Forest plot showing a comparison of follow up Left ventricular end diastolic diameter (LVEDD) between conduction system pacing (CSP) and Biventricular pacing (BiVP) for standard mean difference using random effects model.

Three studies (total n = 223; CSP: 99, BiVP: 124) reported left ventricular end-diastolic diameter outcomes. CSP was associated with a significant reduction in LVEDD, with a pooled SMD of −0.54 (95% CI: 0.82 to −0.27, *p* = 0.0001), consistent with enhanced reverse remodelling. Moderate heterogeneity was observed (*I* ^*2*^ = 59%), likely reflecting differences in baseline ventricular dimensions, pacing modality (HBP vs. LBBAP), and follow-up duration. Despite this, the overall effect remained robust and clinically meaningful, reinforcing the potential of CSP to improve structural outcomes in non-LBBB heart failure cohorts Figure 6.

**FIGURE 7:**
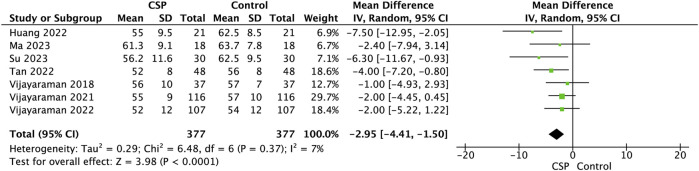
Forest plot showing a comparison of follow-up left ventricular end diastolic diameter (LVEDD) between baseline and follow-up conduction system pacing (CSP) implant for mean difference using a random effects model.

Seven studies comprising 377 patients evaluated the change in LVEDD after CSP implantation. The pooled mean difference was −2.95 mm (95% CI: 4.41 to −1.50, *p* < 0.0001), indicating a significant reduction in ventricular size and suggesting favourable reverse remodelling. The test for overall effect yielded a Z-score of 3.98, confirming statistical significance Figure 7. Heterogeneity was low (I^2^ = 7%), with a Chi^2^ value of 6.48 (df = 6, *p* = 0.37) and Tau^2^ = 0.29, indicating consistent findings across studies. The narrow confidence intervals and low heterogeneity strengthen the reliability of the observed effect.

### NYHA functional class improvement

**FIGURE 8:**
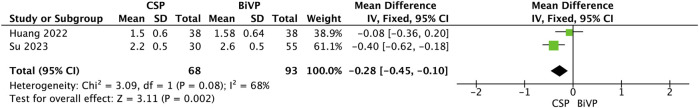
Forest plot showing a comparison of follow up NYHA between conduction system pacing (CSP) and Biventricular pacing (BiVP) for mean difference using fixed effects model.

The comparison of New York Heart Association functional class between conduction system pacing and biventricular pacing was evaluated in two studies comprising 161 patients (68 CSP, 93 BiVP). The meta-analysis revealed a significant reduction in NYHA class favoring CSP, with a pooled mean difference of −0.28 (95% confidence interval [CI], −0.45 to −0.10; *P* = 0.002). This indicates that patients receiving CSP experienced a modest but meaningful improvement in symptoms and functional status compared to those treated with BiVP Figure 8.

Despite moderate heterogeneity (I^2^ = 68%, *P* = 0.08), the overall effect suggests a consistent trend towards better clinical outcomes with CSP, reinforcing its potential as an effective pacing strategy to alleviate heart failure symptoms.

**FIGURE 9:**
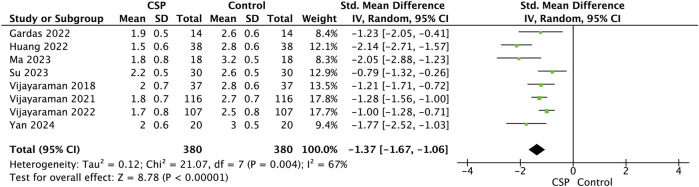
Forest plot showing a comparison of follow up NYHA between baseline and follow-up conduction system pacing (CSP) implant for standard mean difference using random effects model.

In a pooled analysis of studies evaluating New York Heart Association (NYHA) functional class before and after conduction system pacing (CSP) in the same cohort of 380 patients, there was a significant improvement in symptoms following CSP. The meta-analysis showed a standardized mean difference (SMD) of −1.37 (95% CI, −1.67 to −1.06), indicating a substantial reduction in NYHA class, consistent with clinically meaningful symptomatic relief (*Z* = 8.78, *P* < 0.00001) Figure 9.

Although moderate heterogeneity was present (I^2^ = 67%, τ^2^ = 0.12, χ^2^ = 21.07, *df* = 7, *P* = 0.004), the direction and magnitude of effect were consistent across studies, demonstrating that CSP is associated with marked improvement in functional capacity within the same patients.

### Effect on QRS duration

Three studies comprising 223 patients (99 CSP, 124 BiVP) evaluated QRS duration following pacing. The pooled analysis demonstrated a significantly narrower QRS duration with CSP compared to BiVP, with a standardised mean difference (SMD) of −0.99 (95% confidence interval [CI], −1.27 to −0.70; *Z* = 6.57, *P* < 0.00001). This reflects a large effect size favouring CSP in achieving more physiological ventricular activation.

Individual studies consistently supported this finding. Huang et al. (2022) reported the most pronounced difference (SMD = −1.88), while Chen et al. (2023) and Tan (2022) observed substantial reductions (SMDs = −1.13 and −0.96, respectively). Despite variation in magnitude, all studies favoured CSP.

**FIGURE 10:**
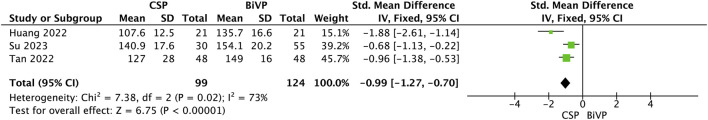
Forest plot showing a comparison of follow up QRS duration between conduction system pacing (CSP) and Biventricular pacing (BiVP) for standard mean difference using fixed effects model.

Moderate heterogeneity was present (I^2^ = 73%, χ^2^ = 7.38, *df* = 2, *P* = 0.02), suggesting variability in baseline QRS morphology or pacing protocols across cohorts. Nonetheless, the consistent direction and strength of effect support the superiority of CSP in preserving native conduction and minimizing electrical dyssynchrony Figure 10 *.*

**FIGURE 11:**
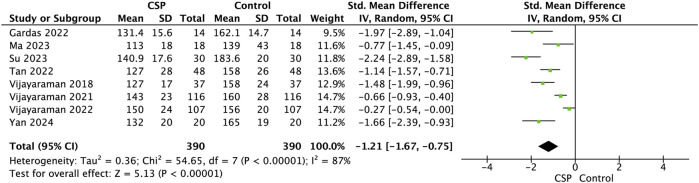
Forest plot showing a comparison of follow up QRS duration between baseline and follow-up conduction system pacing (CSP) implant for standard mean difference using random effects model.

Eight studies (Gardas 2022; Ma et al., 2023; Su 2023; Tan 2022; Vijayaraman 2018, Vijayaraman et al., 2021, Vijayaraman et al., 2022; and Tam et al., 2024), including a total of 780 patients (CSP: 390; Control: 390), were analysed to assess the change in QRS duration following CSP. The pooled analysis demonstrated a statistically significant reduction in QRS duration post-CSP implantation. The overall standardised mean difference (SMD) was −1.21 (95% CI: 1.67 to −0.75, *p* < 0.00001), indicating a large effect size and substantial narrowing of QRS complexes Figure 11.

Among individual studies, Su 2023 reported the most pronounced reduction (SMD = −2.24, 95% CI: 2.89 to −1.58), followed by Gardas 2022 (SMD = −1.97) and Tam et al., 2024 (SMD = −1.66). All studies showed a reduction in QRS duration, with seven of eight studies reaching statistical significance. Vijayaraman 2022 showed a modest but borderline significant effect (SMD = −0.27, 95% CI: 0.54 to −0.00).

Heterogeneity across studies was high (I^2^ = 87%), with a Chi^2^ value of 54.65 (df = 7, *p* < 0.00001) and Tau^2^ = 0.36, indicating substantial variability in effect sizes. This may be attributed to differences in baseline QRS duration, pacing techniques (e.g., His-bundle vs. left bundle branch pacing), or patient characteristics. Despite this, the direction of effect was consistently favourable across all studies. The test for overall effect yielded a Z-score of 5.13, confirming the statistical significance of the pooled result.

### HF hospitalisation and all-cause mortality

**FIGURE 12:**
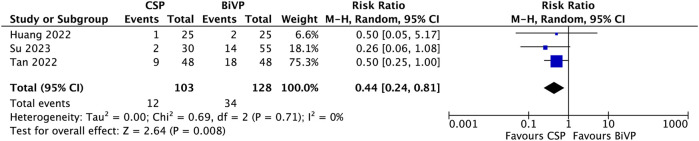
Forest plot showing HF admission between CSP and Biventricular pacing.

In this meta-analysis examining heart failure (HF) hospitalisation outcomes between conduction system pacing (CSP) and biventricular pacing (BiVP), data were pooled from three prospective studies: Huang et al. (2022), Chen et al. (2023), and Tan et al. (2023), encompassing 103 patients in the CSP treatment arm and 128 patients in the BiVP treatment arm. The aggregated risk ratio (RR) for HF hospitalisation significantly favoured CSP, with a pooled RR of 0.44 (95% CI: 0.24–0.81), corresponding to a 56% relative reduction in risk compared to BiVP (Z = 2.64, P = 0.008). Individual study estimates varied, with Tan et al. (2023) contributing the majority of statistical weight (75.3%) and reporting an RR of 0.50 [0.15–1.67], while Huang et al. (2022) and Su et al. (2020) reported RRs of 0.25 [0.05–1.17] and 0.40 [0.09–1.83], respectively. Importantly, statistical heterogeneity was negligible (I^2^ = 0%, Tau^2^ = 0.00, Chi^2^ = 0.69, P = 0.71), suggesting consistency across study-level effects Figure 12. With respect to all-cause mortality, only one study, Tan et al. (2023), provided evaluable data. In this cohort, five of 28 patients in the CSP group died, compared to 19 of 48 in the BiVP group, indicating a numerically lower mortality rate with CSP. While these findings are promising, the mortality analysis is limited by its single-study origin and should be interpreted with caution pending further validation in larger, multicentre trials.

### Procedural-related complications

Procedural safety reporting was variably described among the included studies (Vijayaraman et al., 2021). reported procedural outcomes in 227 successful implantations encompassing both LBBAP and BiVP. Within this cohort, there were three cases of pneumothorax, two device-related infections, and seven instances of lead dislodgement. However, the study did not specify whether these complications occurred in the LBBAP or CRT subgroups (Gardas et al., 2024). documented a single case of lead dislodgement following HBP. Among the remaining nine studies, two explicitly stated the absence of procedure-related complications, while the others did not provide specific information on procedural safety events.

### Methodological quality and risk of bias assessment

Among the included studies, randomised and propensity-matched analyses such as those by Vijayaraman et al., Ma et al., and Upadhyay et al. were judged to have low risk of bias. The majority of observational comparative studies were rated as moderate risk due to retrospective allocation and residual confounding despite adjustment. Single-arm series, including those by Bednarek et al. and Gardas et al., were judged to have a serious risk of bias because of non-consecutive recruitment, absence of comparators, and lack of statistical adjustment. Overall, most of the available evidence carried a moderate risk of bias (Supplementary Table S1).

## Discussion

This meta-analysis yielded four principal findings as follows: (1) conduction system pacing was associated with a significant improvement in left ventricular ejection fraction compared with biventricular pacing. (2) CSP resulted in a reduction in left ventricular end-diastolic diameter, reflecting favourable reverse remodelling. (3) CSP produced meaningful QRS narrowing, indicating superior electrical synchrony. (4) CSP was linked to improvements in New York Heart Association functional class, suggesting symptomatic and functional benefit. (5) CSP was associated with a significantly lower risk of heart failure hospitalisation and a numerically reduced all-cause mortality compared to BiVP.

It is well established that BiVP confers clinical benefit in patients with heart failure and electrical dyssynchrony, with the most significant benefit observed among those with LBBB morphology and QRS duration of ≥150 m (Sipahi et al., 2012). Accordingly, current guidelines designate BiVP as a Class I indication in this population (Glikson et al., 2021). However, the therapeutic efficacy of BiVP in non-LBBB subgroups, including patients with right bundle branch block, and intraventricular conduction delay, remains a matter of controversy. Meta-analyses of large randomised controlled trials (RCTs) consistently demonstrate that patients with RBBB and left ventricular systolic dysfunction do not derive significant benefit from BiVP (Cunnington et al., 2015). In this context, BiVP may induce iatrogenic dyssynchrony, leading to low response rates and, in some cases, increased mortality (Ruschitzka et al., 2013). Thus, non-LBBB conduction morphology is recognised as an unfavourable predictor of CRT response (Cunnington et al., 2015; Zareba et al., 2011).

CSP, through His-bundle pacing and left bundle branch area pacing, has emerged as an alternative strategy for cardiac resynchronisation (Upadhyay et al., 2019; Lustgarten et al., 2015). These techniques achieve more physiological activation of the ventricles, thereby enhancing electrical synchrony, promoting reverse remodelling, improving cardiac function, and ultimately reducing morbidity and mortality compared with BiVP. Permanent HBP is feasible and safe, particularly in patients with RBBB and CRT indications (Sharma et al., 2018). Similarly, Huang et al. pioneered proximal left bundle pacing, demonstrating larger reductions in QRS duration compared with BiVP and effects comparable to HBP. Left ventricular septal pacing has also been shown to produce acute hemodynamic improvement, underscoring the physiological advantage of capturing the native conduction system (Huang et al., 2017).

Despite these promising results, there are currently no large-scale RCTs specifically addressing patients with non-LBBB heart failure. Much of the available evidence is derived from observational or single-arm studies (Sharma et al., 2018; Huang et al., 2020), many of which predominantly enrolled patients with LBBB, with non-LBBB subgroups forming a minority (Vijayaraman et al., 2021; Wu et al., 2021; Pujol-Lopez et al., 2022). In our meta-analysis, the pooled analysis of 13 studies, encompassing 526 patients, CSP improves systolic function, promotes reverse remodelling, enhances functional status, and narrows QRS duration more effectively than BiVP.

### Improvement in left ventricular systolic function

#### LVEF response to CSP compared with BiVP

The pooled analysis demonstrated a significant improvement in LVEF with CSP compared to BiVP, with a mean difference of 5.83% (95% CI, 3.06 to 8.60; Z = 4.12; *p* < 0.0001). This indicates that, on average, patients receiving CSP achieved nearly a 6% higher LVEF at follow-up relative to those treated with BiVP. The superiority of CSP is likely attributable to its ability to restore near-normal electrical activation through recruitment of the His–Purkinje system, which BiVP cannot fully replicate.

#### Pre- and post-implant LVEF with Conduction system pacing

Across 11 studies encompassing 480 patients, CSP implantation resulted in an average absolute LVEF increase of +8.91%. This improvement supports the concept that CSP not only prevents pacing-induced dyssynchrony but may also facilitate reverse remodelling in patients with impaired systolic function. Although moderate heterogeneity was observed, this variability likely reflects differences in patient selection, baseline ventricular function, and pacing modality. Studies reporting greater improvement often included patients with wider QRS duration or higher right ventricular pacing burden at baseline, who might derive greater benefit from resynchronisation via CSP. Importantly, despite these methodological and population-level differences, the uniform direction of effect underscores the robustness of CSP’s impact on ventricular performance. These findings align with emerging clinical evidence that physiological pacing can achieve or even surpass the functional recovery seen with conventional CRT in selected patients, positioning CSP as a promising therapeutic strategy for both bradycardia and heart failure populations.

#### Subgroup analyses of HBP and LBBAP

Both HBP and LBBAP demonstrated notable improvements in left ventricular systolic function, supporting the growing evidence that conduction system pacing can restore physiological ventricular activation. In our pooled analyses, HBP was associated with a significant increase in LVEF from baseline, reflecting its capacity to engage the native His–Purkinje network directly. Similarly, LBBAP showed a mean improvement of approximately 8%, with consistent benefits observed across studies despite moderate heterogeneity. While both approaches achieve electrical and mechanical synchrony superior to conventional right ventricular pacing, LBBAP may offer a more practical alternative in terms of lead stability and procedural success, particularly in patients with distal conduction disease or challenging His capture thresholds. Taken together, these findings reinforce the clinical value of conduction system pacing as a strategy to preserve or recover ventricular function, with LBBAP emerging as a promising evolution of physiological pacing for broader clinical applicability.

Individual studies corroborate the above findings. Vijayaraman et al. (2021) reported a significant increase in LVEF from 33% to 44% following LBBAP in patients with reduced baseline function. Bednarek et al. (2023) observed that patients with baseline LVEF <50% experienced meaningful improvement in both LVEF and global longitudinal strain after LBBAP. Ma et al. (2023) demonstrated substantial echocardiographic and clinical responses in patients with severely reduced LVEF (<30%). The REVERSE trial has further shown that patients with RBBB or IVCD exhibit minimal benefit from BiVP, with LVEF improvements as low as 0.9%–7.2%, compared with robust responses in LBBB (Gold et al., 2012). Collectively, the present evidence strongly supports CSP as a superior resynchronisation modality in restoring systolic function among non-LBBB patients, even in those with profound baseline dysfunction.

### Reverse remodelling and structural outcomes

Structural remodelling, as measured by changes in LVEDD, was another area where CSP demonstrated favourable effects. Across seven pooled studies, CSP implantation resulted in an average reduction of nearly 3 mm in LVEDD, reflecting meaningful reverse remodelling. This aligns with observations from (Gardas et al., 2024), who found reductions in indexed LV volumes after HBP, and from (Chen et al., 2023), who reported sustained reductions in LV size with LOT-CRT compared with BiVP over 24 months. The structural benefits of CSP were also evident in the RBBB cohort studied by Vijayaraman et al. (2022), where reduced ventricular dimensions paralleled improvements in LVEF, despite only modest QRS narrowing. These findings highlight that CSP does more than acutely improve contractility; it promotes durable reverse remodelling by restoring physiologic ventricular activation. This is, in contrast with BiVP, where the ENHANCE-CRT trial and prior RCTs reported limited or inconsistent remodelling in non-LBBB patients. The ENHANCE-CRT trial, which randomised 248 non-LBBB patients to QLV-guided versus anatomically guided LV lead placement, reported a 70% response rate based on composite clinical score, without significant differences between RBBB and IVCD subgroups (Singh et al., 2020).

### Electrophysiologic outcomes: QRS duration

With respect to QRS duration, the degree of narrowing achieved with CRT has been shown to correlate with clinical and echocardiographic outcomes (Bazoukis et al., 2020). Prior studies have established that prolonged QRS duration in IVCD carries an increased risk of arrhythmic death (Tiosano et al., 2016), while greater QRS narrowing after CRT is linked to improved long-term survival (Jastrzębski et al., 2019). Electrical synchrony, reflected in QRS narrowing, was a central mechanistic advantage of CSP over BiVP. The pooled analysis demonstrated a large effect size for QRS reduction (SMD –1.21), with consistent direction of benefit across all eight contributing studies. Chen et al. (2023) reported the most marked reduction in QRS duration with LOT-CRT compared to BiVP, while Tam et al. (2024) showed that personalised ECGi-guided CSP implantation yielded superior reductions in total ventricular activation time, strongly predicting echocardiographic response. Vijayaraman et al. (2021), Vijayaraman et al. (2022) demonstrated that both LBBAP and HBP could achieve QRS narrowing, even in difficult cohorts such as those with RBBB, with reductions correlating with improved clinical outcomes. By contrast, large BiVP trials such as REVERSE study reported minimal QRS narrowing and poor echocardiographic response in IVCD or RBBB subgroups, a finding consistent with our pooled evidence that non-LBBB morphology predicts poor response to BiVP. The consistent QRS shortening achieved with CSP likely explains the superior remodelling and functional outcomes observed.

### Symptomatic and functional improvement

Symptomatic improvement, assessed by the NYHA functional class, also favoured CSP. The pooled analysis demonstrated a large and statistically significant effect size (SMD –1.46), indicating substantial functional recovery following CSP implantation. Comparative studies revealed a modest but consistent advantage of CSP over BiVP, with Tan et al. (2023) showing significantly fewer hospitalisations and improved survival among CSP recipients. In long-term observational cohorts, such as Su et al. (2020) in atrial fibrillation patients undergoing AV node ablation, durable improvements in NYHA class were maintained over 3 years. Similarly, Vijayaraman et al. (2022) found that patients with RBBB and heart failure who underwent LBBAP improved significantly in NYHA class despite only modest QRS shortening, reinforcing the clinical value of CSP even when electrical effects are less dramatic.

### Safety of CSP

The procedural safety profile observed across the included studies suggests that conduction system pacing is generally safe and well tolerated, with complication rates comparable to those reported in conventional pacing or BiVP procedures. The majority of adverse events were minor and manageable, such as lead dislodgement, pneumothorax, or device-related infection. In the largest cohort reported by Vijayaraman et al. (2021) These complications occurred infrequently and within expected ranges for device implantation, although subgroup attribution to LBBAP or BiVP was not specified. Similarly, isolated lead dislodgement was reported in the study by Gardas et al. (2024), underscoring that lead stability remains a technical consideration, particularly during early experience or in His bundle pacing cases. Notably, several studies either reported no complications or did not provide detailed procedural data, which limits the precision of pooled safety estimates. Nevertheless, the overall evidence indicates that with appropriate operator experience and lead selection, CSP can be performed with a safety profile comparable to standard pacing techniques, supporting its wider adoption in clinical practice.

### Why strict LBBB benefits while non-true LBBB may not

The apparent contradiction between favourable CSP effects in our non-LBBB cohort and prior reports of poorer outcomes in patients labelled as LBBB who do not fulfil “Strauss” criteria can be explained by underlying conduction substrates. The Strauss definition identifies complete LBBB with longer QRS thresholds and lateral lead notching, correlating with a proximal His Purkinje block that is highly reversible by resynchronisation therapies (Strauss et al., 2011). In contrast, non-LBBB and many IVCD morphologies often reflect heterogeneous distal Purkinje or intramyocardial conduction delay, scar-related slowing, or mixed activation patterns (Tan et al., 2020). Observational and meta-analytic data demonstrate that patients meeting typical LBBB criteria have a greater CRT response than those with non-LBBB (Saplaouras et al., 2025). Moreover, haemodynamic studies suggest that patients with IVCD exhibit less QRS shortening and attenuated reverse remodelling, even with advanced resynchronisation strategies. These mechanistic differences support a gradient of “resynchronisability,” with typical LBBB showing the greatest benefit, intermediate responses in RBBB amenable to conduction recruitment, and limited benefit in IVCD or non-LBBB.

### Future perspectives

The findings underscore the need for future research and adjustments in clinical practice. First, well-powered trials are crucial to see if improvements in surrogate markers with CSP lead to fewer heart failure hospitalisations and deaths, including long-term benefits. Second, research should identify which non-LBBB subgroups benefit most from CSP, considering individual differences like conduction issues and myocardial features, possibly using imaging or electrocardiographic predictors for better patient selection. Third, advances in technology are vital for wider CSP use, addressing current implantation challenges with device innovation and standard procedures to improve safety and success. Fourth, guideline integration requires consensus as more data emerge, potentially positioning CSP as a first-line or rescue strategy. Lastly, CSP’s impact on healthcare systems deserves attention; if proven superior to BiVP, it could be more cost-effective by lowering hospitalisations and complications, necessitating resource, efficiency, and outcome evaluations for policy development.

### Limitations

Several limitations should be considered. Many included studies were observational and had a relatively small population size, introducing potential selection bias. The follow-up duration varied, limiting conclusions regarding long-term outcomes, such as mortality or sustained remodelling. Substantial heterogeneity was observed in some pooled analyses, likely reflecting differences in baseline patient characteristics, pacing technique, and study design. Moreover, QRS narrowing, while supportive, remains a surrogate marker and does not directly capture clinical outcomes such as survival or hospitalisation. These factors highlight the need for cautious interpretation, though the consistent direction of effect across diverse settings is reassuring. An important methodological limitation is that some of our pooled analyses of baseline versus follow-up outcomes in single-arm cohorts used SMDs without paired correlation, effectively treating pre- and post-measures as independent. This approach is not ideal and may yield artificially narrow confidence intervals. Although sensitivity analyses using reported change scores produced consistent directions of effect, the magnitude of improvement should be interpreted cautiously. We therefore stress that single-arm pre–post findings are exploratory, whereas our principal conclusions are derived from direct comparative data between CSP and BiVP.

## Conclusion

In patients with heart failure and non-LBBB conduction patterns, CSP provides significant improvements in systolic function, ventricular remodelling, symptom burden, and electrical synchrony compared with BiVP, with additional evidence suggesting reduced heart failure hospitalisation and a potential survival advantage. These findings suggest that CSP is a promising physiological pacing method, particularly for a patient group that has historically shown poor outcomes with standard BiVP. While further high-quality randomised trials are required, CSP represents a significant advance in the management of non-LBBB heart failure and a potential key development in resynchronisation therapy.

## Data Availability

The original contributions presented in the study are included in the article/Supplementary Material, further inquiries can be directed to the corresponding author.
