# Supplementary material for: Efficacy and safety of conduction system pacing in heart failure patients with non-left bundle branch block morphology: a systematic review and meta-analysis
Source: Front Physiol. 2025 Nov 17;16:1716337. doi: 10.3389/fphys.2025.1716337 (PMC12665559; doi:10.3389/fphys.2025.1716337)

| **Study** | **Were There Clear Inclusion and Exclusion Criteria?** | **Was There Clear Reporting of Patient Demographics?** | **Were Comparator Groups Similar and Recruited from Same Population?** | **Were the Exposures Measured Similarly Across Groups?** | **Were Exposures Measured in a Valid and Reliable Way?** | **Were Confounding Factors Identified?** | **Were Strategies to Deal with Confounding Factors Stated?** | **Were Participants Free of the Outcome at the Start?** | **Was Follow-up Complete and Long Enough?** | **Was Outcome Assessment Valid and Reliable?** | **Overall Risk of Bias** |
| --- | --- | --- | --- | --- | --- | --- | --- | --- | --- | --- | --- |
| Vijayaraman et al. | No (non-consecutive series) | Yes | N/A (single-arm) | N/A | Yes (standard implantation protocols) | Partially (non-randomized, clinical discretion) | No (observational) | Yes (follow-up post-implantation) | Partially (7.8 months, some remote follow-ups) | Yes (echocardiography, NYHA class, biomarkers) | Moderate (due to selection and confounding bias) |
| Vijayaraman et al. | Yes (CRT non-responders with ‚â•6 months follow-up) | Yes | Yes (LBBAP vs BVP groups) | Yes | Yes (device interrogation, echo) | Yes (differences in CRT indication, baseline LVEF) | Partially (baseline characteristics reported, no matching or PS adjustment) | Yes | Partially (mean 12¬±9 months, retrospective) | Yes (EF, NYHA, device metrics) | Moderate to Serious (due to potential residual confounding) |
| Su et al. | Yes (defined criteria for baseline LBBB and CRT indication) | Yes | Partially (LBBAP vs BVP, but no randomization) | Yes | Yes (standard implantation protocol) | Yes (baseline clinical and ECG differences noted) | Partially (multivariable adjustment) | Yes | Yes (median follow-up 12 months) | Yes (LVEF, NYHA, QRS duration) | Moderate (due to non-randomized design and residual confounding) |
| Vijayaraman et al. (PACE-Randomized Pilot Subanalysis) | Yes (AVN ablation candidates with AF and HFrEF) | Yes | Yes (HBP vs BVP, though this is a pilot) | Yes | Yes (standard procedural techniques) | Yes (baseline clinical differences acknowledged) | Yes (randomization, even if open-label) | Yes | Yes (12-month follow-up completed) | Yes (EF, NYHA, QOL, 6MWT) | Low |
| Tan et al. | Yes (patients with HFrEF undergoing CRT) | Yes | Partially (LBBAP vs BVP, retrospective design) | Yes | Yes (standardized implantation procedures) | Yes (acknowledged in baseline comparisons) | Partially (multivariate and sensitivity analysis) | Yes | Yes (median 12 months) | Yes (echo, NYHA, clinical outcomes) | Moderate (retrospective design, potential residual confounding) |
| Jastrzebski et al. | Yes (patients undergoing LBBAP with sufficient follow-up) | Yes | N/A (single-arm study) | N/A | Yes (standard protocol, LBBAP verification) | Partially (observational without control) | No (no comparator or adjustment) | Yes | Yes (6-month clinical and echocardiographic follow-up) | Yes (EF, NYHA, ECG parameters) | Serious (due to single-arm design and lack of adjustment) |
| Huang et al. | Yes (LBBB, HFrEF, CRT indication) | Yes | Partially (LBBAP vs BVP, retrospective allocation) | Yes | Yes (standard implantation criteria and ECG confirmation) | Yes (reported differences in baseline ECG and medications) | Partially (PS matching used) | Yes | Yes (median 18.5 months) | Yes (LVEF, clinical outcomes, ECG) | Moderate (retrospective design with attempts at adjustment) |
| Chen et al. (Multicenter comparison study) | Yes (patients with heart failure and LBBB indicated for CRT) | Yes | Partially (LBBAP vs BVP, center-dependent allocation) | Yes | Yes (standard criteria for pacing capture and lead placement) | Yes (baseline LVEF and NYHA differences noted) | Partially (multivariable Cox regression used) | Yes | Yes (mean 17 months follow-up) | Yes (clinical and echocardiographic assessment) | Moderate (retrospective design with center-level variability) |
| Vijayaraman et al. (Real-world multicenter registry) | Yes (patients undergoing LBBAP with CRT indication) | Yes | Yes (LBBAP vs BVP, matched across centers) | Yes | Yes (standardized capture criteria, pacing parameters) | Yes (noted in baseline demographics) | Partially (adjusted analyses reported) | Yes | Yes (12-month clinical follow-up with device and echo data) | Yes (EF, QRS, NYHA, hospitalization) | Moderate (observational nature despite multicenter data and adjustment) |
| Vijayaraman et al. (Early feasibility and safety analysis) | Yes (patients with heart failure indicated for LBBAP) | Yes | N/A (single-arm prospective registry) | N/A | Yes (implantation criteria and electrical parameters) | Partially (baseline clinical characteristics reported) | No (no comparator or statistical adjustment) | Yes | Partially (3-month follow-up for safety endpoints) | Yes (procedure success, safety, lead parameters) | Serious (single-arm short-term design with no adjustment) |
| Huang et al. | Yes (patients with heart failure and CRT indication) | Yes | Yes (BVP vs LBBAP in a real-world setting) | Yes | Yes (standard CRT criteria and invasive hemodynamic measurement) | Yes (baseline group differences acknowledged) | Partially (matched analysis and adjusted comparisons) | Yes | Partially (acute hemodynamic response endpoints) | Yes (LV dP/dtmax, QRS duration, ECG and device data) | Moderate (despite objective endpoints, acute design limits generalizability) |
| Ma et al. | Yes (HFrEF, LBBB patients undergoing CRT) | Yes | Yes (LBBAP vs BVP) | Yes | Yes (standardized ECG and echo protocols) | Yes (baseline group differences reported) | Yes (propensity score matching and adjustment) | Yes | Yes (median 12.5 months) | Yes (LVEF, NYHA, clinical events) | Low |
| Yan et al. | Yes (patients with CRT indication and LBBB) | Yes | Partially (LBBAP vs BVP, center-based allocation) | Yes | Yes (standard implantation and ECG criteria) | Yes (baseline clinical and ECG characteristics reported) | Partially (multivariate regression used) | Yes | Yes (12-month follow-up) | Yes (EF, NYHA, hospitalization, QRS duration) | Moderate (non-randomized, adjusted analysis performed) |
| Gardas et al. | Yes (patients receiving LBBAP with CRT indication) | Yes | N/A (single-arm study) | N/A | Yes (electrophysiological criteria and imaging) | Partially (noted in limitations) | No (no comparator group or statistical adjustment) | Yes | Yes (6-month and 12-month outcomes assessed) | Yes (QRS duration, EF, NYHA, lead parameters) | Serious (non-comparative single-arm design with no adjustment) |
| Ma et al. (LVAT-based LBBAP optimization study) | Yes (patients with LBBAP and HFrEF receiving CRT) | Yes | Yes (LVAT-optimized vs non-optimized) | Yes | Yes (standard LVAT protocol, ECG, and echo) | Yes (group differences and effect modifiers identified) | Partially (multivariate analysis applied) | Yes | Yes (6-month clinical follow-up) | Yes (EF, NYHA, echo-derived parameters) | Moderate (non-randomized, single-center, adjusted analysis) |
| Vijayaraman et al. | Yes (patients undergoing conduction system pacing with CRT indication) | Yes | Yes (HBP vs BVP) | Yes | Yes (implant success, pacing thresholds, QRS duration) | Yes (clinical differences noted between groups) | Partially (multivariable adjustment used) | Yes | Yes (12-month clinical and device follow-up) | Yes (clinical outcomes, echocardiography, hospitalization) | Moderate (non-randomized comparison with adjustments) |
| Huang et al. (Physiological CRT vs Conventional CRT) | Yes (HF patients undergoing physiological CRT including HBP, LBBAP, or BiVP) | Yes | Partially (group assignment based on procedural feasibility) | Yes | Yes (implant protocols and echocardiography) | Yes (baseline ECG and functional differences reported) | Partially (multivariable Cox and subgroup analyses applied) | Yes | Yes (up to 24 months) | Yes (death, hospitalization, LVEF, NYHA) | Moderate (retrospective nature and group imbalance) |
| Upadhyay et al. | Yes (patients with LBBB undergoing CRT, excluding secondary cardiomyopathy) | Yes | Yes (HBP vs BVP in matched settings) | Yes | Yes (standardized criteria for HBP and BVP) | Yes (noted differences in CRT type, QRS duration, and baseline EF) | Yes (inverse probability treatment weighting used) | Yes | Yes (median 19.1 months) | Yes (composite clinical outcome, EF, QRS duration) | Low |

Supplementary Table 1. Risk of bias assessment of included randomised and observational studies using RoB 2 and ROBINS-I tools, respectively

Supplementary Table 1. Risk of bias assessment of included randomised and observational studies using RoB 2 and ROBINS-I tools, respectively

CRT = cardiac resynchronization therapy; LVEF = left ventricular ejection fraction; HFrEF = heart failure with reduced ejection fraction; LBBB = left bundle branch block; LBBAP = left bundle branch area pacing; BVP = biventricular pacing; HBP = His bundle pacing; AVN = atrioventricular node; AF = atrial fibrillation; QOL = quality of life; 6MWT = six-minute walk test; NYHA = New York Heart Association; EF = ejection fraction; ECG = electrocardiogram; LVAT = left ventricular activation time

Supplementary figures

1. Funnel plot of LVEF comparing CSP and BiVP


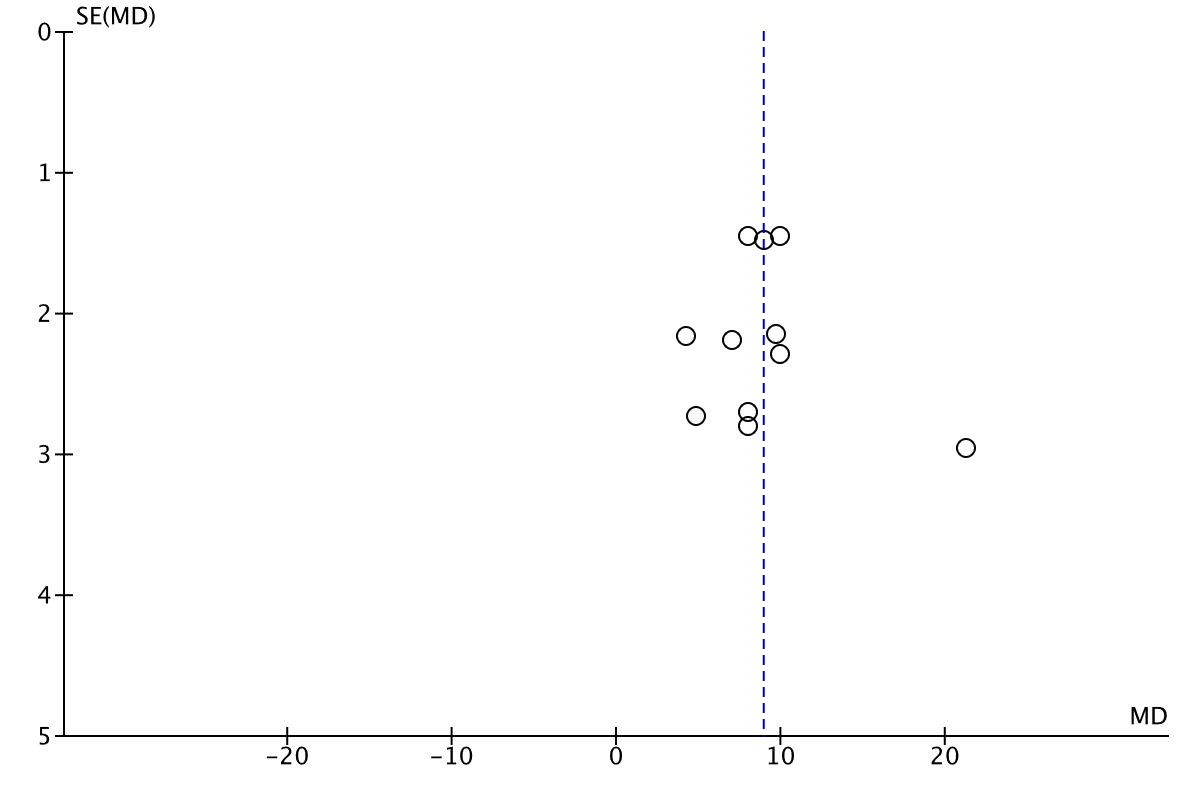


1. Funnel plot of LVEF comparing baseline and follow-up CSP


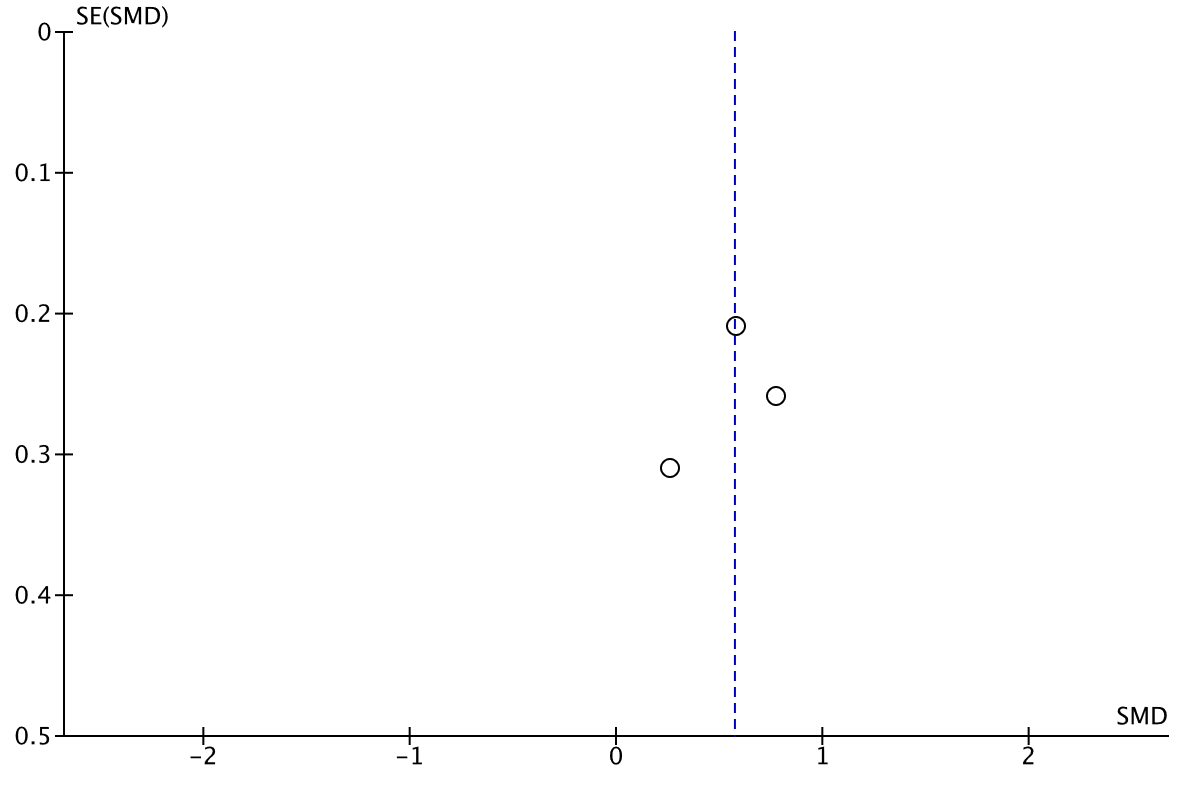


1. Funnel plot of LVEDD comparing CSP vs BiVP


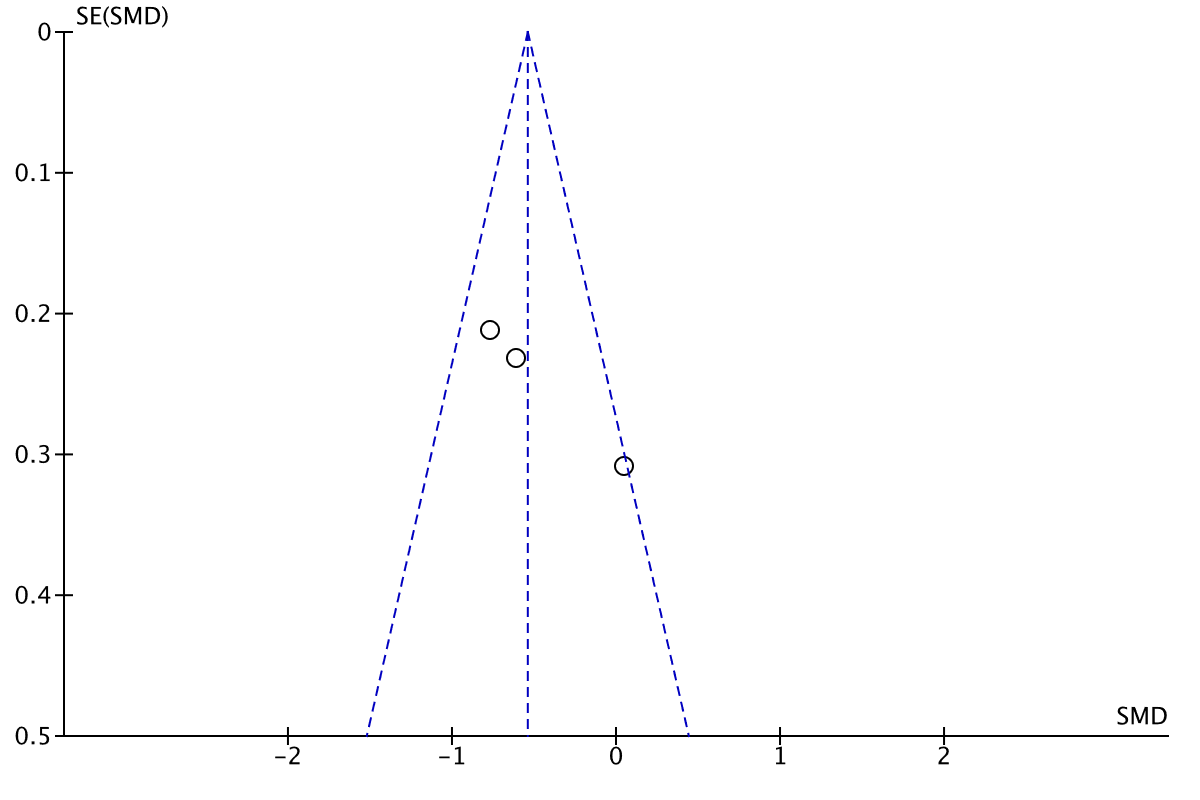


1. Funnel plot of LVEDD comparing baseline and follow-up CSP


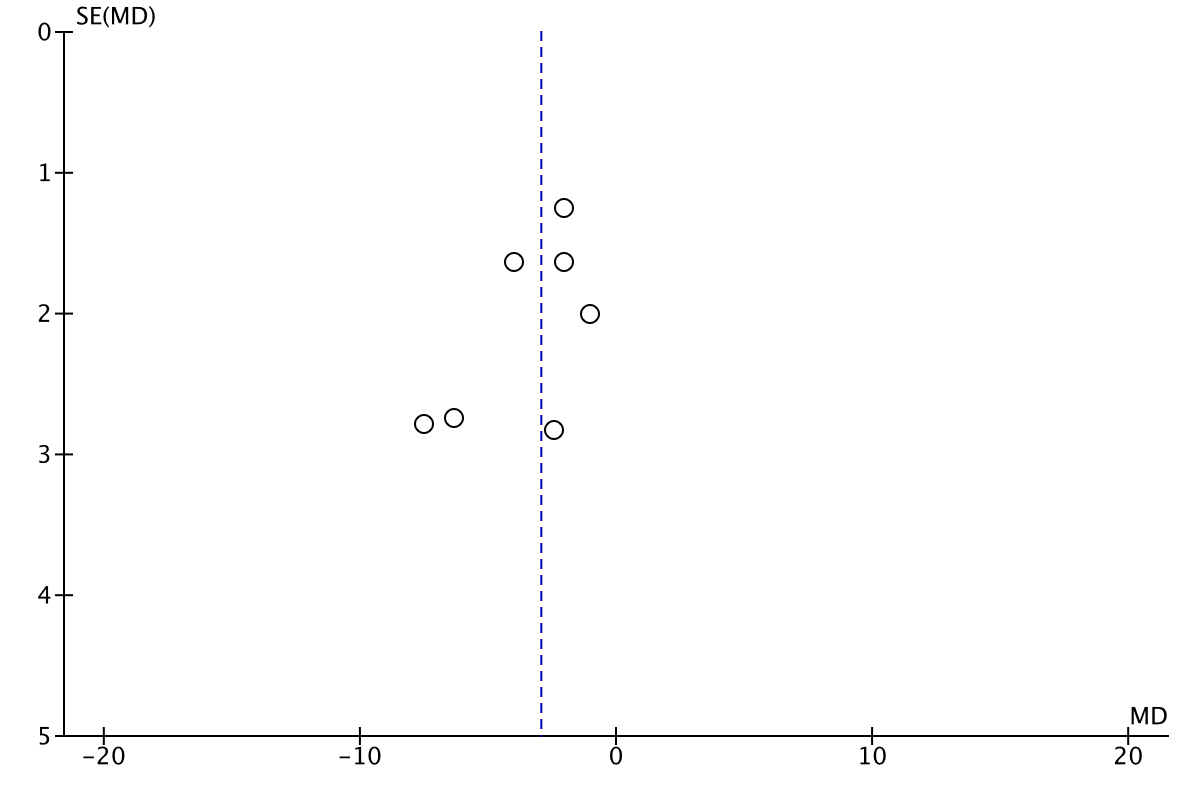


1. Funnel plot of NYHA comparing CSP vs BiVP


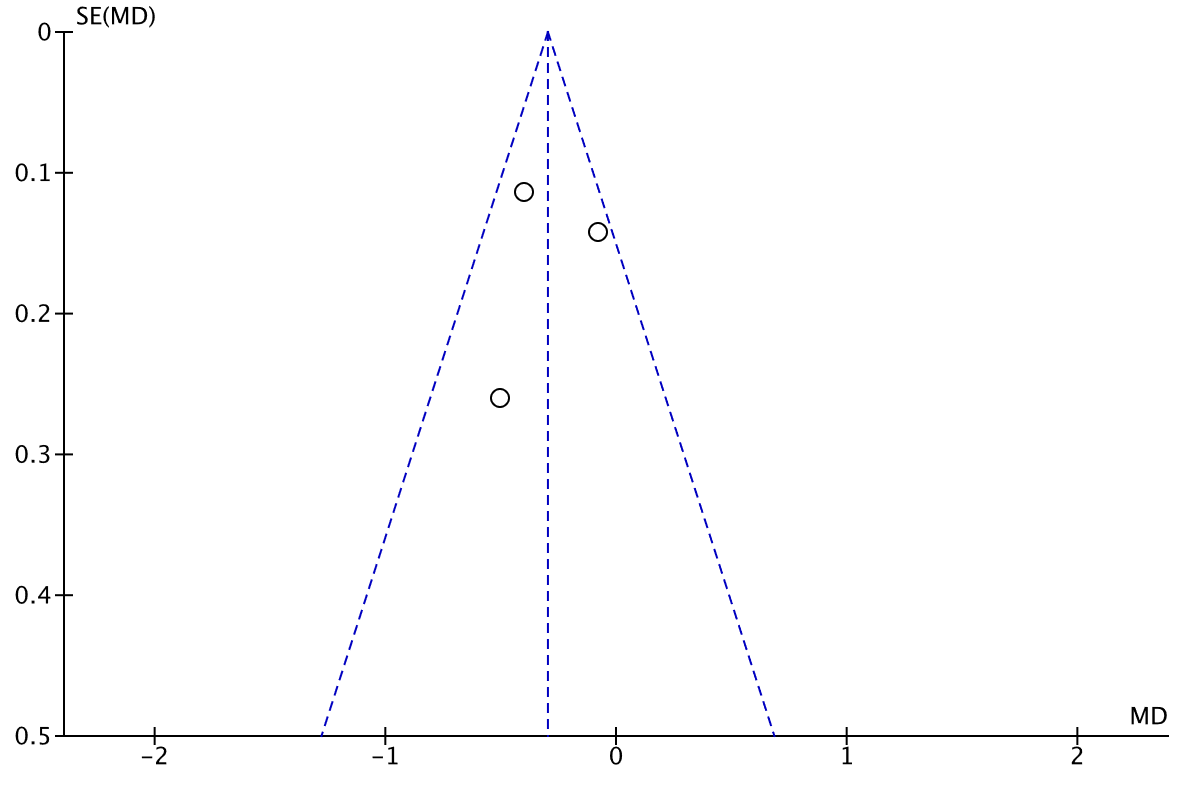


1. Funnel plot of NYHA comparing baseline and follow-up CSP


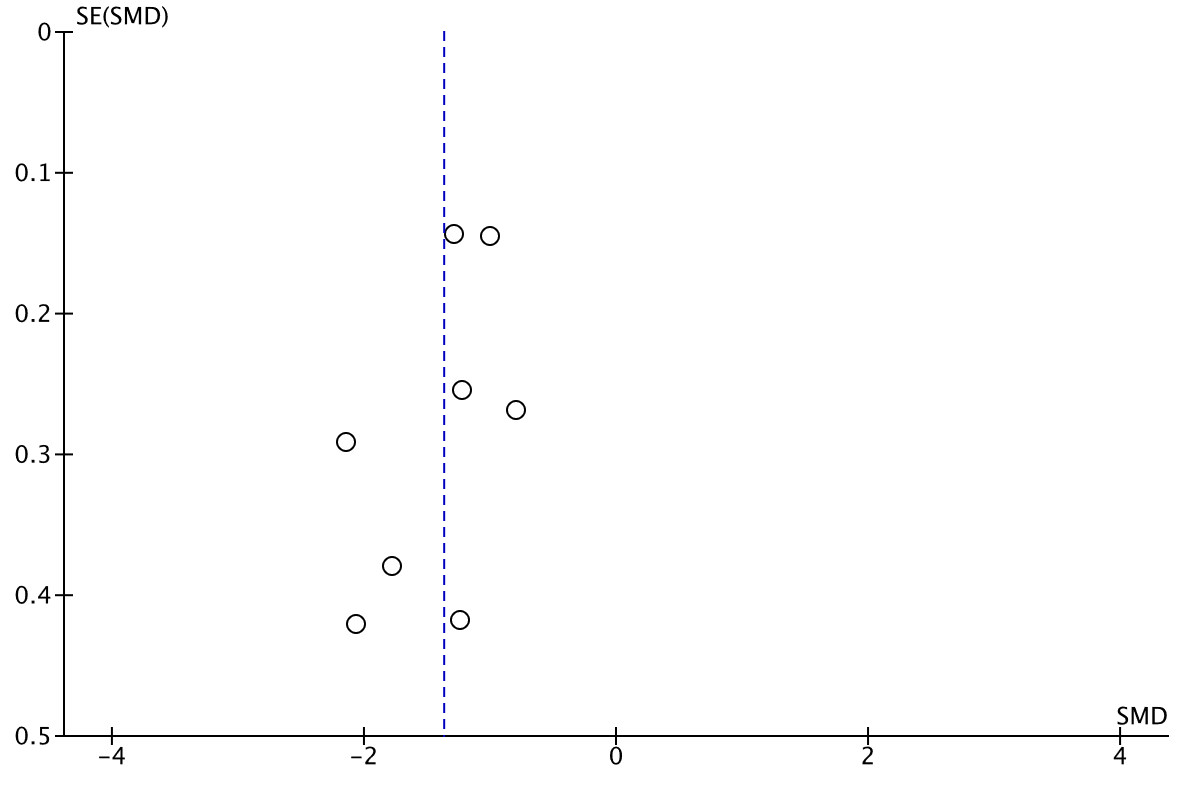


1. Funnel plot of QRS duration comparing CSP vs BiVP


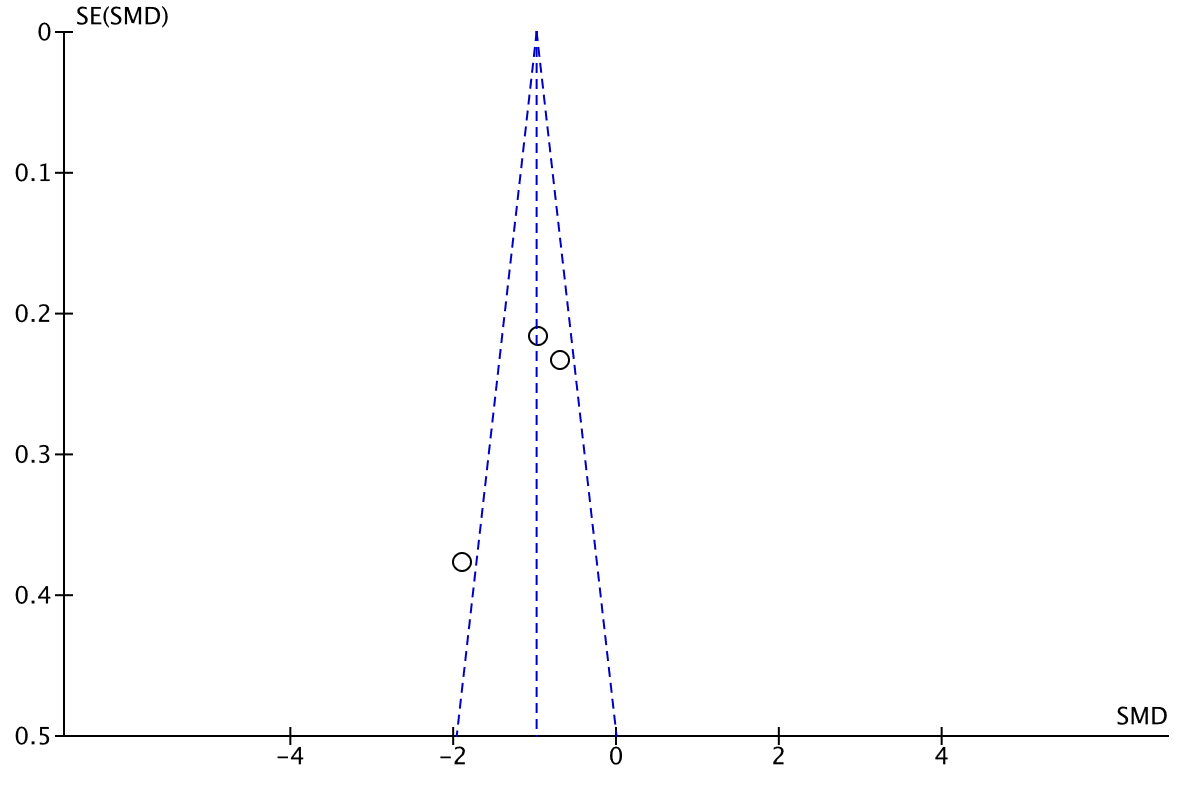


1. Funnel plot of QRS duration comparing baseline and follow-up CSP


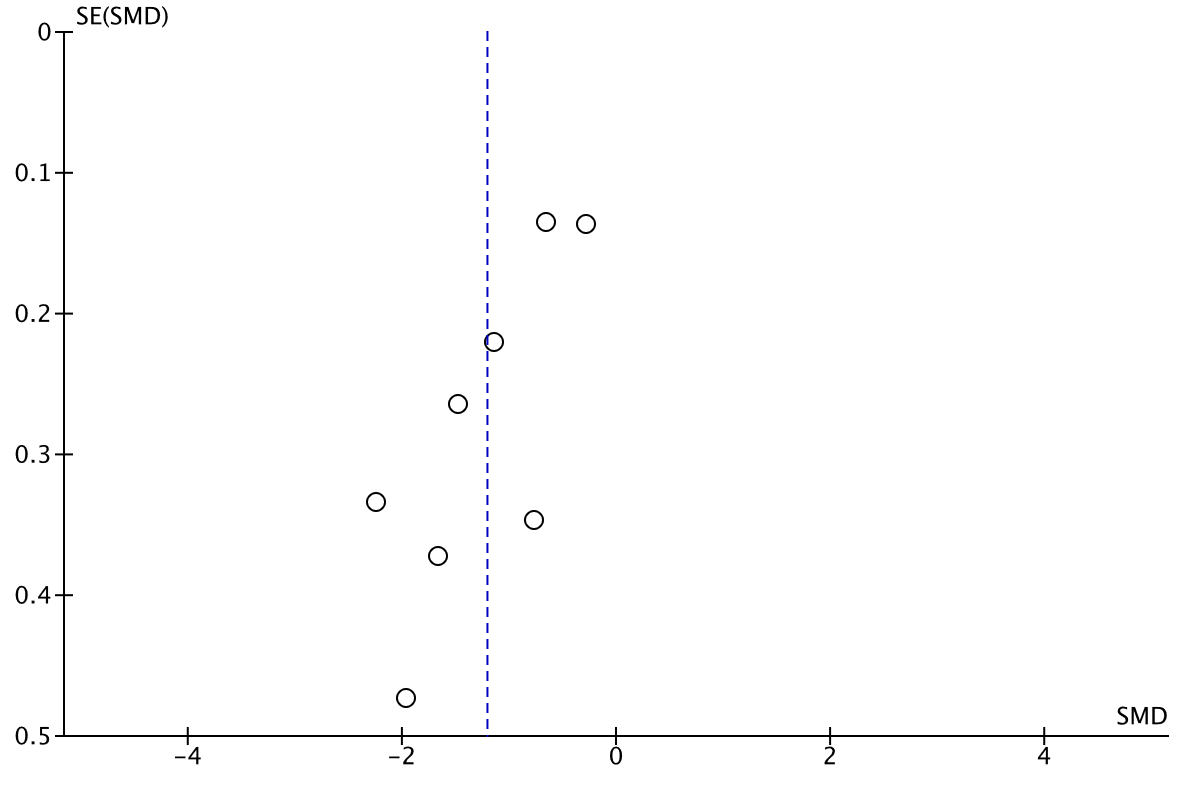

Supplement: Supplementary file 1 [file DataSheet1.docx]
